# Nutrient‐Synbiotic Complex Ameliorates LPS‐Induced Depressive‐Like Behavior via Modulation of Gut Microbiota and Tryptophan Metabolism in Mice

**DOI:** 10.1002/fsn3.70628

**Published:** 2025-07-15

**Authors:** Zhipeng Liu, Shengchao Shi, Xiaoyu Zhang, Chao Wu, Qin Yang, Simeng Ren, Yujuan Shan, Guanqiong Na

**Affiliations:** ^1^ School of Public Health Wenzhou Medical University Wenzhou China; ^2^ Cixi Biomedical Research Institute Wenzhou Medical University Cixi China; ^3^ Department of Psychology, College of Liberal Arts Wenzhou‐Kean University Wenzhou China

**Keywords:** depression, gut microbes, nutrients, prebiotics, probiotics

## Abstract

The conventional antidepressant treatments are ineffective, having undesirable side effects and relapse susceptibility. Nutrient and probiotics supplementation may have adjuvant antidepressant effects. This study aims to develop and assess the antidepressant properties of a nutrient‐synbiotic complex and investigate the underlying mechanisms. We randomly divided into control, model, and four complex groups (nutrient, prebiotic, probiotic and united all components, respectively). We used lipopolysaccharide (LPS) to establish the depression model and assessed depressive‐like behaviors using various behavioral tests, neuroplasticity, tryptophan pathway metabolites, and brain‐derived neurotrophic factor (BDNF) in serum and prefrontal cortex (PFC). Intestinal microbiome analysis was performed using 16S rRNA gene sequencing. The nutrient‐synbiotic complex presented beneficial effects against the depression‐like behaviors induced by LPS, mitigated the increased level of kynurenine, and decreased levels of 5‐hydroxytryptamine and BDNF both in serum and PFC (*p* < 0.05). It also reduced neuronal damage in PFC after LPS exposure (*p* < 0.05). Additionally, the nutrient‐synbiotic complex effectively prevented the disturbance in abundance and diversity of the intestinal flora induced by LPS (Chao1: *p* = 0.013, ACE: *p* = 0.006, PCoA: *R* ^2^ = 0.25, *p* = 0.003). A total of nine bacterial genera were negatively associated with depression, while eight were positively associated with depression. Among them, *g__Family_XIII_UCG_001*, *g__Clostridia_vadinBB60_group*, and *g__Lactobacillus* showed significant intergroup differences (*p* < 0.05). This is the first study to demonstrate that a combined nutrient‐synbiotic complex can prevent depressive‐like behavioral changes induced by LPS. The underlying mechanisms may be associated with modifying the composition of the gut microbial community and improving the tryptophan metabolic homeostasis.

## Graphical abstract

Complex interventions significantly prevent the depression‐like behaviors. Nutrient‐synbiotic complex presents the more beneficial effects. Nutrient‐synbiotic complex modifies the composition of gut microbial community. Nutrient‐synbiotic complex improves the tryptophan metabolic homeostasis.

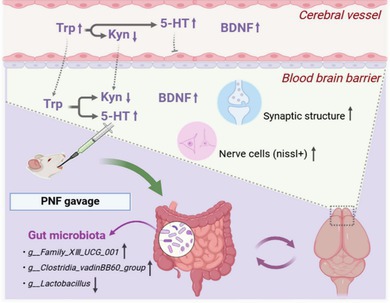

## 1 Introduction

Depression, a prevalent mood disorder, ranks as the primary cause of disability on a global scale (Chen et al. 2024). At present, depression affects over 350 million individuals worldwide (Herrman et al. 2022). Despite this, effective remedies for depression remain scarce, and conventional medications have proven to be ineffective, benefiting only a minority of depressed patients and having undesirable side effects and relapse susceptibility (Feng et al. 2024). Depression sufferers require treatments that are both safe and effective immediately. Given that dietary nutrients were found to be associated with depression, dietary factors may be a viable option (Ekinci and Sanlier 2023).

Dietary interventions can mildly alleviate depressive symptoms, according to a meta‐analysis of 16 studies focusing on non‐clinical populations (Firth et al. 2019), which may be closely related to the nutrients in diets. For example, folate, vitamin B_6_, and vitamin B_12_, which are implicated in neurological development and functional refinement, have the potential to mitigate depressive‐like behaviors and thereby reduce the risk of developing depression (Abdelmaksoud et al. 2019; Kris‐Etherton et al. 2021). Zinc supplementation significantly improves depressive‐like behavior and reduces the risk of developing depression (Wang et al. 2018). Furthermore, higher levels of omega‐3 fatty acids, tryptophan, iron, and selenium intake are also negatively associated with the risk of depressive disorder (Chen et al. 2021; Li et al. 2018; Lin et al. 2010). Thus, correction of nutritional deficiencies and supplementation with psychosocial‐related nutrients are crucial in the prevention and treatment of depression.

Probiotics, which are live microorganism strains, confer health benefits to the host when administered in appropriate quantities. Probiotic strains supplementation, including *Lactobacillus acidophilus* , *Lactobacillus casei* , and *Bifidobacterium longum* , was found to ameliorate depressive symptoms in clinical studies (Jach et al. 2023). Furthermore, the antidepressant efficacy of probiotics may be enhanced through a synergistic effect resulting from the interaction of multiple strains, suggesting that the rational use of multi‐strain probiotics might yield superior results compared to the use of single strains (Chapman et al. 2012). Prebiotics, the substrates that can be selectively utilized by gut microbiota, are advantageous to the health of hosts and promote the growth of numerous beneficial bacteria. Similarly, probiotic supplementation, whether taken alone or in combination, has been shown to be an effective treatment for depression (Liu et al. 2023). Among them, oligofructose (FOS) and oligogalactose (GOS) are the prebiotics that are most frequently employed in the therapeutic management of depression.

In light of the growing evidence that substantiates the potential utility of dietary interventions as an adjunctive treatment for mental disorders, we developed and validated the antidepressant efficacy of a nutrient‐synbiotic complex. While nutrients and probiotics show individual efficacy, their synergistic effects on depression remain unexplored. Our results demonstrated that the nutrient‐synbiotic complex significantly attenuated the depression‐like behaviors and neurons injured by LPS.

## 2 Materials and Methods

### 2.1 Preparation of Nutrient‐Synbiotic Complex

The nutrient‐synbiotic complex comprises the following primary ingredients: six probiotic strains ( *B. longum* , *L. casei* , *Lactobacillus suis*, *Lactobacillus rhamnosus* , *Lactobacillus fermentum* and *Lactococcus lactis subspecies lactis*) produced by Run Ying Bioengineering (Shanghai) Co. Ltd.; two prebiotics (oligogalactose and oligofructose) produced by Quantum High‐Tech (Guangdong) Biological Co. Ltd., and a nutrient complex (vitamin B_12_, folic acid, selenium, iron and zinc) formulated by Shuo Wei Nutritional Technology (Guangzhou) Co. Ltd. The intake dosage of the nutrient‐synbiotic complex was determined in accordance with B14880‐2012 National Standard for Food Safety, Standard for the Use of Food Nutritional Enrichment.

### 2.2 Animals

The animal experiments were approved by the Animal Ethics Committee of Wenzhou Medical University (Approved No. XMS9022‐0635) and adhered to the Animal Ethics Statement. Specific pathogen‐free (SPF) male ICR mice (18–20 g, 3 weeks old) were procured from Beijing Viton Lihua Laboratory Animal Technology Co. Throughout the duration of the experiment, we kept the mice under SPF laboratory conditions (day/night time allocation of 12 h/12 h, lighting time: 09:00–21:00, temperature: 22°C ± 2°C, humidity: 55% ± 5%). We ensured that all animals had unrestricted access to food and water, and that the potable water was replaced daily. Every 2–3 days, we cleaned the feces and changed the bedding.

### 2.3 Experimental Design and Group

**FIGURE 1:**
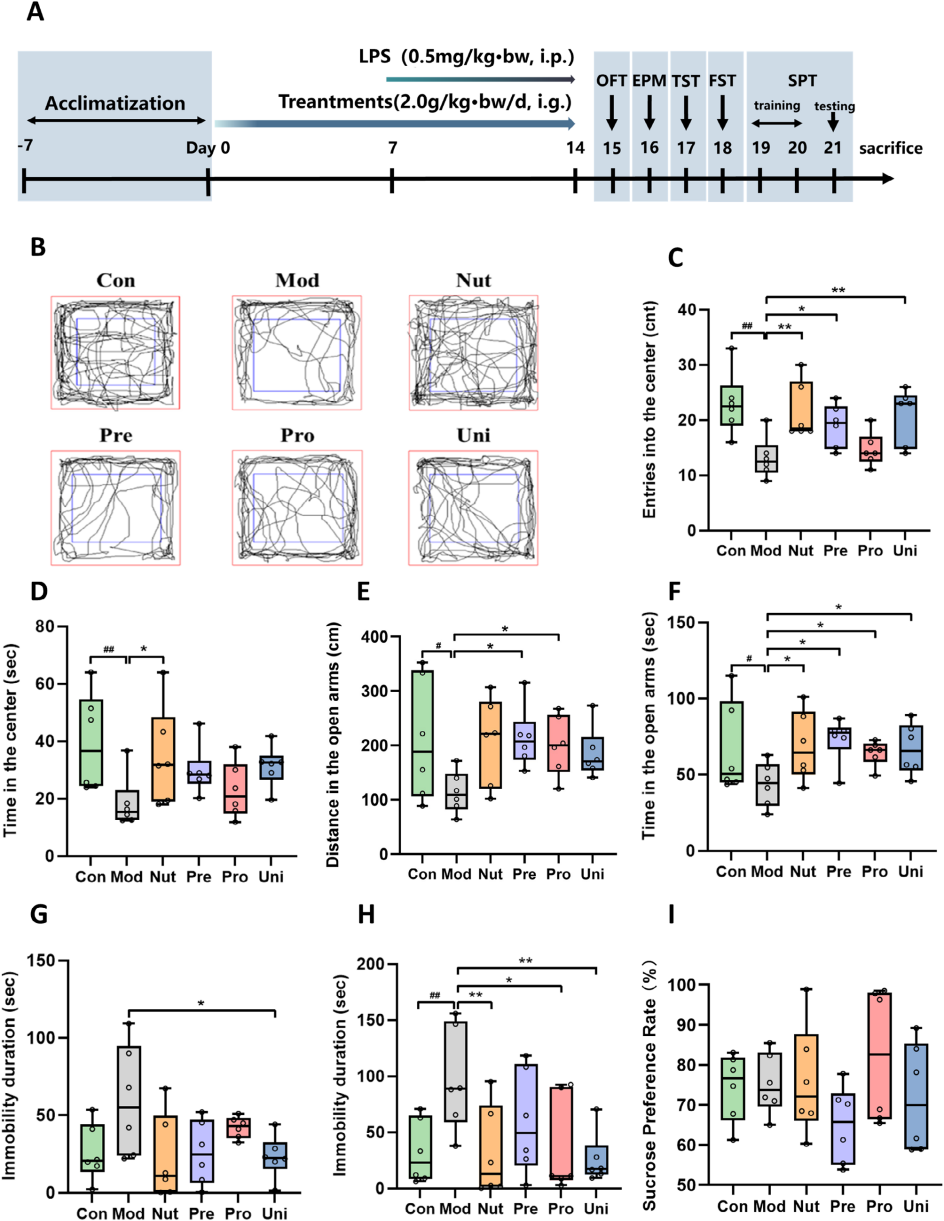
Nutrient‐synbiotic complex alleviates LPS‐induced depressive‐like behaviors. (A) Study design schematic. (B) Representative OFT images depicting the behavioral trajectory of each group. Quantification on entries into the center (C) and time in the center (D) of OFT. Quantification on distance (E) and time (F) in the open arms of EPM, immobility duration in TST (G) and FST (H), and sucrose preference (%) in SPT (I). Data are presented as box and scatter plots with min to max, *n* = 6/group. ^#^ *p* < 0.05 and ^##^ *p* < 0.01 versus the Con; *p < 0.05 and **p < 0.01 versus the Mod. Significance was evaluated by ordinary one‐way ANOVA followed by LSD's multiple comparisons test in (C–I). Con, control group; EPM, elevated plus maze; FST, forced swimming test; LPS, lipopolysaccharide; LSD, least significance difference; Mod, model group; Nut, nutrients group; OFT, open field test; Pre, prebiotics group; Pro, probiotics group; SPT, sucrose preference test; TST, tail suspension test; Uni, united group.

According to body weight, we randomly divided 48 ICR mice into the following groups: control group (Con), model group (Mod), nutrient group (Nut), prebiotic group (Pre), probiotic group (Pro), and united all compounds group (Uni), with eight mice in each group. The number of animals used was determined based on statistical requirements and ethical considerations, ensuring scientifically valid results while minimizing animal use and maintaining welfare standards. Following a 7‐day period of acclimation and feeding, the experimental animals commenced the formal experiment. We administered maltodextrin via gavage to mice in the Con and Mod groups, while providing the respective complex intervention to the Nut, Pre, Pro, and Uni groups (Table S1). Following a 7‐day intervention period, we administered 0.5 mg/kg lipopolysaccharide (LPS) intraperitoneally to Mod and treatment group mice after the 7‐day intervention period to induce depression in the mouse model (He et al. 2020). In contrast, we injected the Con group mice intraperitoneally with sterile saline for seven consecutive days. We justify our use of LPS in Note S1. Throughout the modeling period, we maintained concurrent experimental interventions in all groups. To minimize animal stress, we prioritized gavage before intraperitoneal injections due to the latter's injurious effects. We weighed mice and recorded food consumption every 3 days. We initiated behavioral tests 14 days post‐modeling and outline the full protocol in Figure 1A.

### 2.4 Animal Behavior Test

We evaluated depressive‐like behavior using Open Field Test (OFT), Elevated Plus maze (EPM) Test, Tail Suspension Test (TST), Forced Swimming Test (FST), and Sucrose Preference Test (SPT) as described (Zhao et al. 2023). To minimize carry‐over effects on neuronal plasticity, we randomly selected six mice per group and performed behavioral tests in this sequence: OFT, EPM, TST, FST, and SPT (Figure 1A). We conducted behavioral assessments under controlled lighting conditions with minimal noise disruption. We also cleaned apparatuses with 75% ethanol between animal trials. We conducted all behavioral tests under blinded conditions; testers were unaware of group allocations during scoring. More detail can be found in Note S2.

### 2.5 Samples Collection

After the behavioral tests, we fasted the mice for 12 h, anesthetized them, and collected blood samples through the orbital venous plexus. Then, we centrifuged the blood to obtain serum and stored samples at −80°C. Subsequently, we immediately performed aseptic dissection in a clean bench. We first perfused the left ventricle with phosphate buffer solution (PBS), then rapidly extracted the brain. On a −20°C pre‐chilled plate, we dissected the prefrontal cortex (PFC) into sterile centrifuge tubes. For intestine sampling, we segmented the colon, collected distal fecal pellets into sterile tubes, and harvested other vital organs. After weighing organs and calculating organ‐to‐body weight ratios, we snap‐froze all samples in liquid nitrogen for −80°C storage.

### 2.6 Nissl Staining

We anesthetized the remaining two mice and collected apical blood prior to perfusion of the left ventricle with PBS and fixation with 4% paraformaldehyde solution. After rapidly removing the PFC as demonstrated above, we immediately fixed the left PFC in the 4% paraformaldehyde solution at 4°C for 48 h. We then embedded tissues in paraffin, sectioned them at 5 μm thickness, and stained sections with 0.1% cresyl violet at 37°C for 30 min. Using a computer‐coupled inverted microscope, we captured images of Nissl‐stained sections at 100× and 400× magnifications. As previously described (Jing et al. 2025; Stein‐Behrens et al. 1994), we classified neurons as undamaged when exhibiting intact morphology, visible nuclei, and abundant Nissl's bodies, while identifying damaged cells by their indistinguishable nuclei and dark purple Nissl positivity. Finally, we quantified Nissl‐positive cell counts and synaptic ultrastructural indices using Image J 1.8.0 software.

### 2.7 Examination of Neuronal Structure by Transmission Electron Microscopy (TEM)

We fixed the right PFC samples (volume of 1 mm^3^) in 2.5% glutaraldehyde at 4°C overnight, post‐fixed them in a 1% osmium tetroxide for 1 h, and stained them with uranyl acetate solution for 1 h. We then dehydrated the samples using an acetone gradient series and embedded them in resin blocks. After double‐staining ultrathin sections with 3% uranyl acetate–lead citrate, professional technicians performed transmission electron microscopy (TEM) analysis.

### 2.8 Detection of Key Metabolites

We measured serum and PFC concentrations of tryptophan pathway metabolites (tryptophan [Try], kynurenine [Kyn], 5‐hydroxytryptamine [5‐HT]) and brain‐derived neurotrophic factor (BDNF) using enzyme‐linked immunosorbent assay (ELISA) kits (Shanghai Enzyme‐linked Biotechnology Co. Ltd., Shanghai, China) according to the manufacturer's instructions. Prior to ELISA, we homogenized PFC tissues in ice‐cold PBS (1:9 w/v) and determined total protein concentration with a BCA Protein Assay Kit (Beyotime Biotechnology Co. Ltd., Shanghai, China). We then normalized final concentrations of Try, Kyn, 5‐HT, and BDNF to total protein concentration.

### 2.9 DNA Extraction and16S rRNA Gene Sequencing

Bacterial genomic DNA from fecal samples was extracted using the PowerMax (stool/soil) DNA isolation kit (MoBio Laboratories, Carlsbad, CA, United States) according to the manufacturer's instructions by professional technicians. Subsequently, the quantity and quality of extracted DNA were measured by Nanodrop (Thermo Fisher Scientific, Fair Lawn, NJ, USA) and agarose gel electrophoresis, respectively. The V3‐V4 region of bacterial 16S rRNA gene was amplified. The PCR cycling parameters as follow: initial denaturation at 98°C for 30 s; 30 cycles of denaturation at 98°C for 15 s, annealing at 58°C for 15 s, and extension at 72°C for 15 s; followed by a final extension at 72°C for 1 min. The pair‐end sequencing was performed using the Illlumina NovoSeq6000 platform at GUHE Info Technology Co. Ltd. (Hangzhou, China). The microbiota sequencing data were mainly performed using QIIME2. We performed the species annotation, alpha‐diversity indices (Chao 1 richness estimator, abundance‐based Coverage estimator, Shannon diversity index, and Simpson diversity index) and Beta‐diversity analysis (principal coordinates analysis) of the intestinal flora in QIIME2

### 2.10 Statistical Analysis

We utilized SPSS 26.0 and R 4.1.2 to perform statistical analysis on the study's data and used GraphPad Prism 8.4.3 for data visualization. Unless otherwise stated, we expressed data as “mean ± standard error of deviation”. We conducted the statistical analysis of normally distributed quantitative data with uniform variance using one‐way ANOVA. For post hoc comparisons, we applied the LSD method. Conversely, we compared non‐normal or heterogeneous data using the non‐parametric test Kruskal‐Wallis to identify intergroup differences. We established a significance level of *p* < 0.05 to indicate statistical significance. We analyzed species differences in microbial community 16S sequencing data using nonparametric tests. We screened biomarkers by integrating the nonparametric Kruskal‐Wallis and Wilcoxon rank sum tests with linear discriminant analysis (LDA) to identify significantly different species between groups. We also applied Benjamini‐Hochberg to control for multiple comparisons. Then, we analyzed the predominance of bacterial communities between groups using LEfSe on the Huttenhower Lab Galaxy Server 2.0 (http://galaxy.biobakery.org/). We examined correlations between gut microbial abundance and primary depression‐related indicators using Spearman Correlation Analysis to identify key gut microbes as functional marker flora. We generated the heatmap by ChiPlot platform (https://www.chiplot.online/).

## 3 Results

### 3.1 Nutrient‐Synbiotic Complex Ameliorates LPS‐Induced Depressive‐Like Behavioral Changes in Mice

**FIGURE 2:**
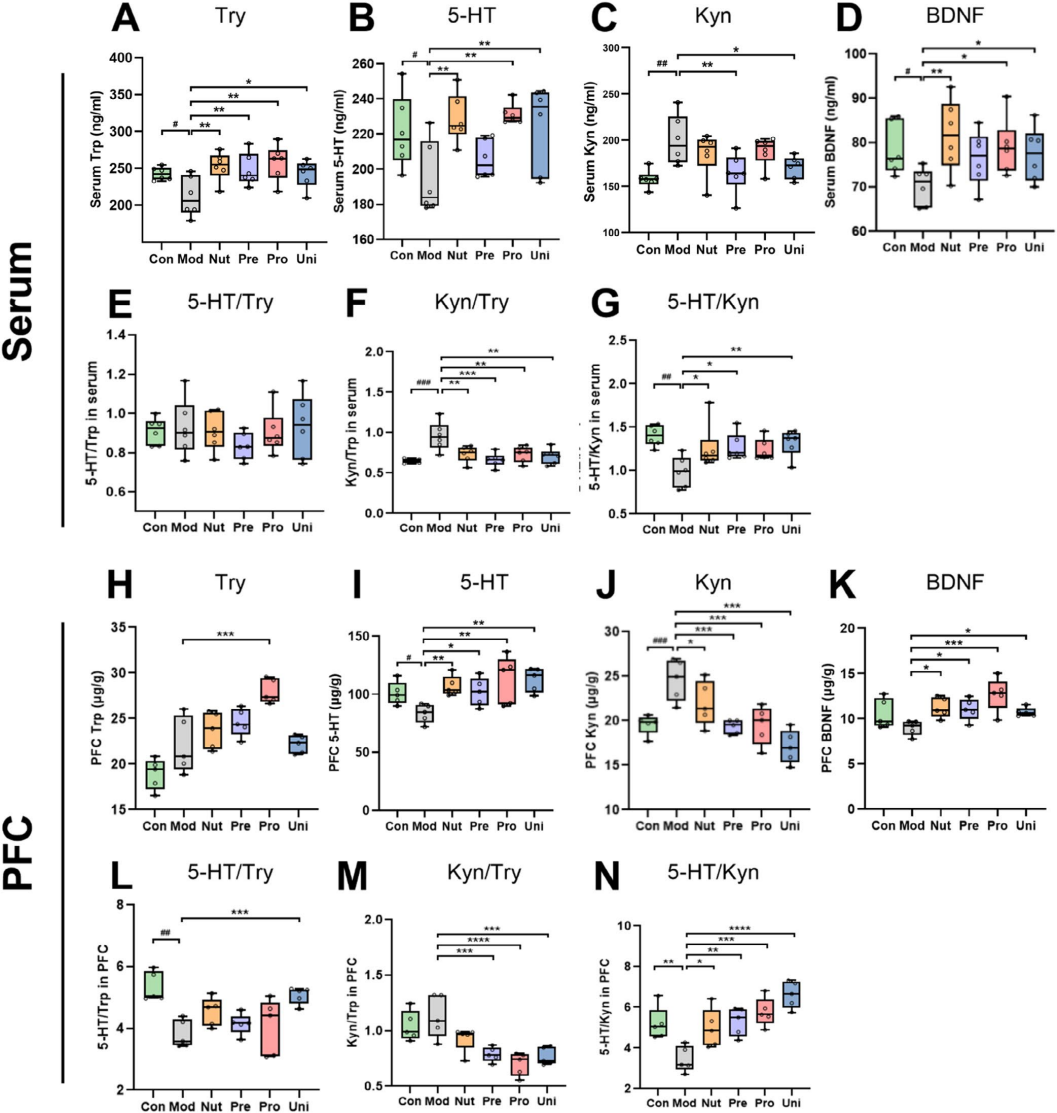
Nutrient‐synbiotic complex alleviates the disruption of neurotransmitter metabolism in the Trp pathway and BDNF in the mice serum and PFC induced by LPS. Variations in depression‐related metabolites are illustrated in the serum (A–G) and PFC (H–N). Data are presented as box and scatter plots with min to max, *n* = 5 or 6/group. ^#^ *p* < 0.05, ^##^ *p* < 0.01, and ^###^ *p* < 0.001 versus the Con; *p < 0.05, **p < 0.01, and ***p < 0.001 versus the Mod. Significance was evaluated by ordinary one‐way ANOVA followed by LSD's multiple comparisons test in (A–N). 5‐HT, 5‐hydroxytryptamine; BDNF, brain‐derived neurotrophic factor; Con, control group; EPM, elevated plus maze; FST, forced swimming test; Kyn, kynurenic; LPS, lipopolysaccharide; LSD, least significance difference; Mod, model group; Nut, nutrients group; PFC, prefrontal cortex; Pre, prebiotics group; Pro, probiotics group; Trp, tryptophan; Uni, united group.

**FIGURE 3:**
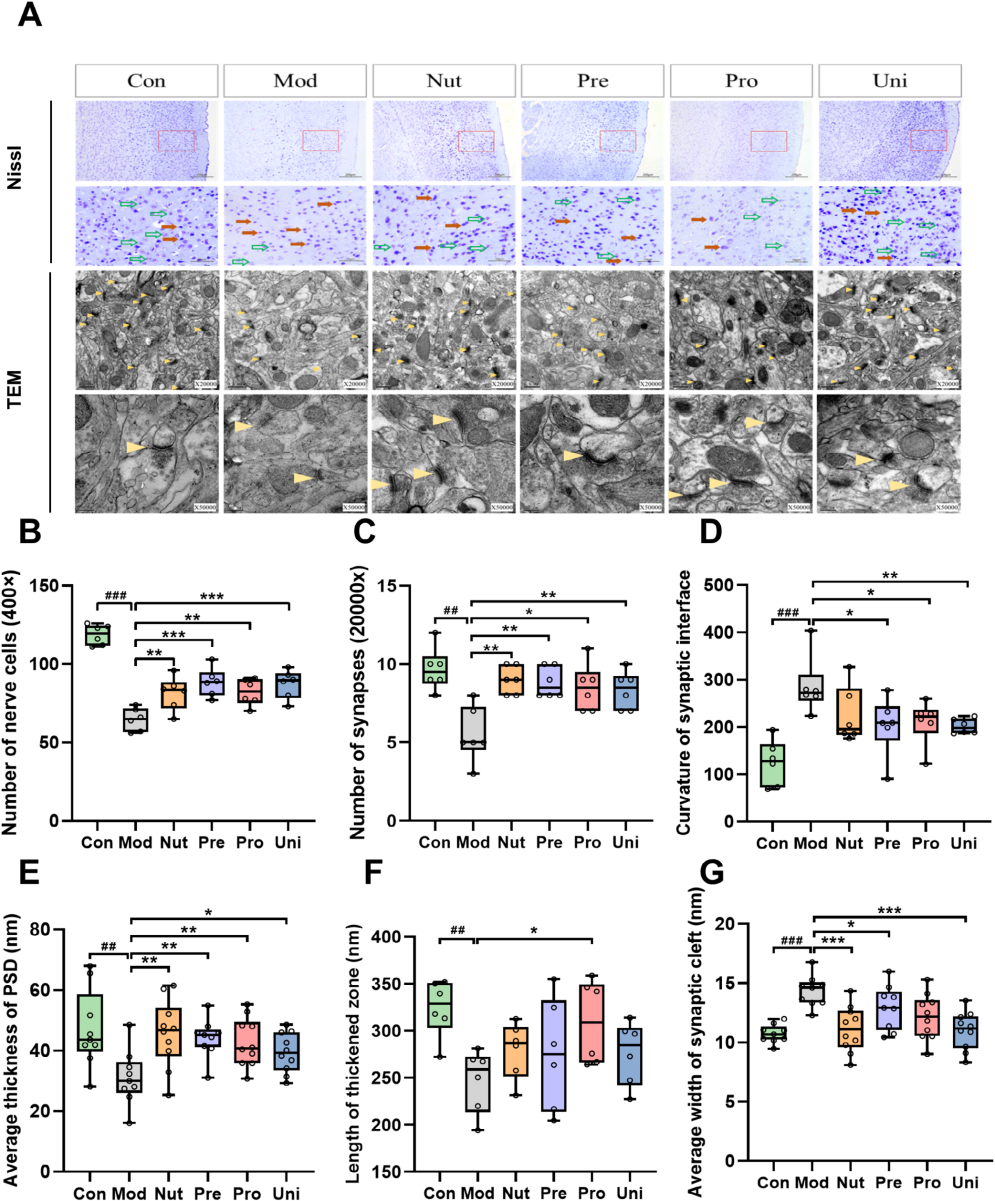
Nutrient‐synbiotic complex treatment ameliorates the neuronal structure alteration in the PFC induced by LPS. (A) Demonstrating neuronal morphology and synaptic plasticity alterations of the PFC. The first two rows of micrographs were using Nissl staining (scales bar = 200 μm and 50 μm, 100× and 400× magnifications), and the remaining micrographs are the ultrastructure of synapses observed by TEM (scales bar = 0.5 μm and 200 nm, magnifications 20,000× and 500,000× magnifications). Six visual fields of the PFC were photographed in each section from 400× magnification of Nissl staining and 20,000× magnifications of TEM, respectively. (B) Quantitative analysis of the numbers of Nissl‐positive neurons in the PFC. (C–G) Quantitative analysis of ultrastructural morphology in PFC neurons. Data are presented as box and scatter plots with min to max, *n* = 5 or 6/group. ^##^ *p* < 0.01 and ^###^ *p* < 0.001 versus the Con; *p < 0.05, **p < 0.01, and ***p < 0.001 versus the Mod. Significance was evaluated by ordinary one‐way ANOVA followed by LSD's multiple comparisons test in (B–G). Con, control group; LPS, lipopolysaccharide; LSD, least significance difference; Mod, model group; Nut, nutrients group; PFC, prefrontal cortex; Pre, prebiotics group; Pro, probiotics group; PSD, postsynaptic density; TEM, transmission electron microscope; Uni, united group.

Firstly, we monitored changes in body weight and food intake of the mice throughout the experiment. The LPS treatment resulted in a reduction in body weight and appetite in the mice (*p* < 0.05). However, when compared to the Mod group, there were no significant differences observed in the complex intervention groups (*p* > 0.05, Figure S1). Likewise, the impacts of LPS‐induced and complex intervention on the majority of organ coefficients were insignificant (*p* > 0.05, Figure 2). Then, we employed behavioral tests to investigate the effect of complex intervention on LPS‐induced depressive‐like behavior. Compared to mice in the Con, LPS induction reduced the frequency, duration, and distance of mice entering the central area in the OFT (*p* < 0.05, Figures 1B–D and 3), as well as the residence time and movement distance of mice entering the open arms of EMP (*p* < 0.05, Figures 1E,F and 3). Furthermore, mice in the Mod group showed significantly prolonged immobility time in the FST and TST (*p* < 0.05, Figure 1G,H). However, LPS‐exposed mice did not exhibit pleasure deficit in the SPT (*p* > 0.05, Figure 1I). These results illustrated that mice in the Mod exhibited the core depressive symptoms such as typical anxiety and behavioral despair under LPS exposure. In contrast, Nut, Pre, Pro, and Uni groups exhibited varying degrees of symptomatic relief. Though all complexes demonstrated efficacy in ameliorating depressive‐like behaviors induced by LPS, the nutrient‐synbiotic complex demonstrated a more pronounced amelioration of depressive symptoms.

### 3.2 Nutrient‐Synbiotic Complex Reverses the Imbalance of Tryptophan Metabolism

**FIGURE 4:**
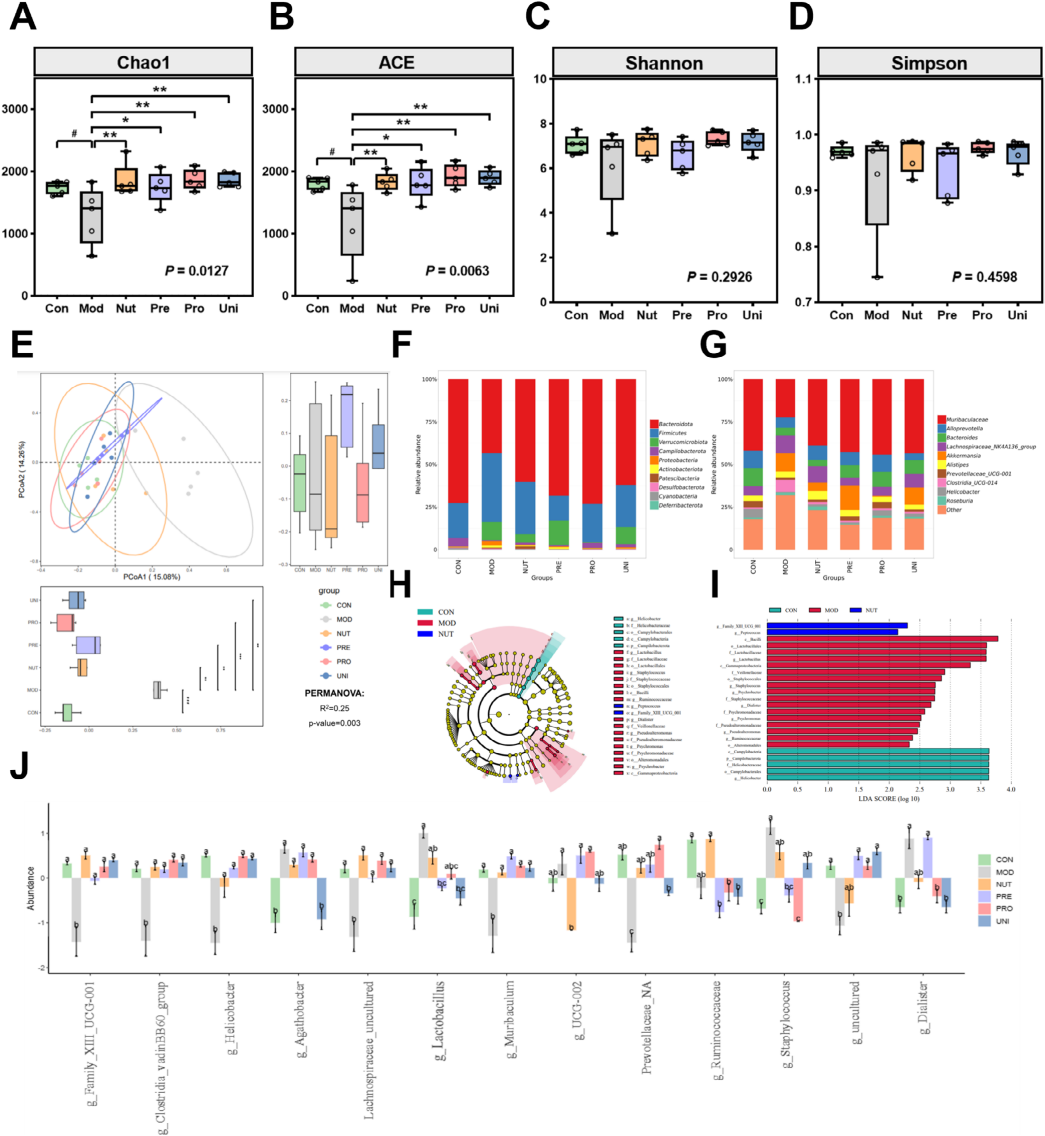
Nutrient‐synbiotic complex regulates the gut microbiome community structures in LPS‐induced mice. (A–D) Alpha‐diversity indexes, including Chao1, ACE, Shannon, and Simpson indexes, stand for the richness and diversity of each group. (E) PCoA analysis based on Bray‐Curtis distance shows the Beta‐diversity of each group at the ASV level. (F, G) The community distribution at phylum and genus levels. (H, I) Cladogram of LEfSe analysis and histogram of LDA value distribution show the differently enriched gut microbiome among different groups. LDA score > 2. (J) Differential gut microbiome based on multiple comparisons at the genus level. Data are presented as box and scatter plots with min to max, *n* = 5 group. ^##^ *p* < 0.01 and ^###^ *p* < 0.001 versus the Con; *p < 0.05, **p < 0.01, and ***p < 0.001 versus the Mod in (A–E). Means denoted by a different letter indicate significant differences between groups (*p* < 0.05) in (J). Significance was evaluated by ordinary one‐way ANOVA followed by LSD's multiple comparisons test in (A–J). ASV, amplicon sequence variant; Con, control group; LDA, linear discriminant analysis; LEfSE, linear discriminant analysis effect size; LPS, lipopolysaccharide; LSD, least significance difference; Mod, model group; Nut, nutrients group; PCoA, principal coordinates analysis; Pre, prebiotics group; Pro, probiotics group; Uni, united group.

Depression and anxiety‐like behavior have been found to be significantly correlated with neurotransmitter metabolism. Tryptophan, being a precursor to neuroactive Kyn and 5‐HT, is particularly significant in the regulation of mood (Note S3 and Figure 4). Following a series of intraperitoneal LPS injections for seven consecutive days, the serum levels of 5‐HT and Trp in the Mod group were significantly decreased compared to those of the Con group (*p* < 0.05). Conversely, Kyn was largely increased (*p* < 0.01, Figure 2A–C), indicating that LPS shifted the metabolic pathway from Trp to Kyn. The increase of Kyn and decrease of 5‐HT in serum were consistent with the relevant molecular characteristics of depression. Only the levels of 5‐HT and Trp in the Uni group were significantly increased, and the level of Kyn was significantly decreased (*p* < 0.05). However, the relief produced by the other complexes was of varying degrees and not universally significant. Moreover, Kyn/Trp in the serum of the Mod mice increased significantly (*p* < 0.001) in comparison to the Con mice, whereas 5‐HT/Kyn decreased significantly (*p* < 0.01, Figure 2E–G). The dysregulation of serum 5‐HT and Kyn ratios was partially reversed in all mice following the complex interventions, with Kyn/Trp exhibiting more pronounced changes.

Given that prefrontal cortex (FPC) activity is critical for emotional processing and is regulated by 5‐HT, we further examined levels of 5‐HT, Kyn, and Trp in the PFC. Not surprisingly, the levels and ratios of three indices were largely consistent with the findings in serum (Figure 2H–N). This provides additional evidence that LPS altered synaptic plasticity, which may contribute to depressive‐like behaviors. Through the complex interventions, this imbalance could be substantially alleviated, and the behavioral changes observed in mice could be reversed.

### 3.3 Nutrient‐Synbiotic Complex Reverses the BDNF Perturbation

BDNF, an increasingly recognized regulator of neuronal plasticity in various brain types, has been associated with antidepressant properties. In this regard, we assessed the fluctuations in BDNF levels in both serum and PFC, the results are illustrated in Figure 2D,K. Compared to mice in the Con, the BDNF levels of serum and PFC were significantly (*p* < 0.05) or tended to decrease in the Mod group. The complex intervention reversed this trend. Thus, the findings indicate that the LPS treatment‐induced deductions in BDNF in serum and PFC of mice were substantially mitigated by all complex interventions. This may be attributable to the complex' potential antidepressant properties.

### 3.4 Protective Effects of the Nutrient‐Synbiotic Complex on PFC Neurons

Progressive synaptic degeneration, neuronal damage, and synaptic loss in the PFC are recognized as significant pathogenic factors in depression (Kang et al. 2012). Neurons of the PFC in the Con were systematically and densely packed, exhibiting conspicuous Nissl bodies (Figure 3A). In contrast, neurons in the Mod displayed atypical characteristics, including aberrant shapes, blurred boundaries, uneven dimensions, irregular and sparse distribution, and a significant reduction in Nissl‐positive neurons (*p* < 0.001). The complex interventions resulted in a significant improvement in the diminution of Nissl‐positive neurons in the mice PFC (*p* < 0.01 or *p* < 0.001, Figure 3B). As shown in Figure 3A and Figure S5, the LPS‐induced treatment has a detrimental impact on various ultrastructural characteristics of the PSD in mice, including the number of synapses, curvature of the posterior membrane, thickness of the PSD, width of the cleft, and length of the thickened area. In comparison to the Con, the number of synapses in the Mod group was considerably diminished (*p* < 0.01), whereas the number of synapses was increased with different complexes (*p* < 0.05, Figure 3C). Furthermore, the synaptic ultrastructure of the PFC was altered in the Mod group compared to Con, which included an increase in the curvature of the posterior membrane (*p* < 0.001, Figure 3D), a reduction in the average and maximal thickness of the PSD (*p* < 0.01, Figure 3E and Figure S6), a decrease in the length of the thickened area (*p* < 0.05, Figure 3F), and a decrease in the average and maximal width of the synaptic cleft (*p* < 0.001, Figure 3G and Figure S6). However, all of these changes were restored by complex interventions. In a word, the findings indicated that depressive‐like behavior induced by LPS in mice could lead to a series of structural alterations in the PFC, including synaptic loss, flatter posterior membrane interface, thinner PSD, shorter thickened area, and wider synaptic gap. These structural changes may be associated with the development of depressive behavior in mice. Furthermore, the complexes may have the potential to reverse the aforementioned synaptic structural changes.

### 3.5 Effects of the Nutrient‐Synbiotic Complex on the Intestinal Flora

Depression has been linked to alterations in the composition of the gut microbiome, which is significantly influenced by diet (Ross et al. 2024). Both the Chao1 index and ACE index values of the Mod were significantly lower than those of the Con, whereas the groups exhibited substantially higher values than the Mod (*p* < 0.05, Figure 4A,B). Conversely, we observed no significant difference between the groups in terms of the Shannon and Simpson index (*p* > 0.05, Figure 4C,D). We identified significant variations in the structure of gut microbial communities between groups by PCoA analysis based on Bray‐Curtis distance (PERMANOVA: *R* ^2^ = 0.25, *p* = 0.003, Figure 4E). Specifically, the PCoA1 and PCoA2 axes accounted for 15.08% and 14.26% of the sample differences, respectively. The figure discernibly differentiated the Mod from the other groups along the PCoA1 axis. We performed abundance analysis of intestinal flora at the phylum and genus levels. *Muribaculaceae* and *Bacteroides*, the dominant genus within the *Bacteroidota* phylum, were less abundant in the Mod, whereas they were significantly enriched in other groups (Figure 4F,G). Using the LEfSe analysis, we identified species with significant differences between groups. A comprehensive assortment of 25 intestinal bacteria, ranging from the phylum to the genus level, exhibited noteworthy variations (Figure 4H,I and Table S2). Multiple comparative analyses of differential gut microbiomes at the genus level revealed that the relative abundance of four bacterial genera (e.g., *g_Lactobacillus, g_Staphylococcus*) increased while the abundance of seven bacterial genera (e.g., *g_Family_XIII_UCG‐001*, g_Clostridia_vadinBB60_group, g_Muribaculum) decreased in the Mod group (Figure 4J and Table S3). Nevertheless, the relative abundance changes induced by LPS were significantly ameliorated by the complex interventions.

**FIGURE 5:**
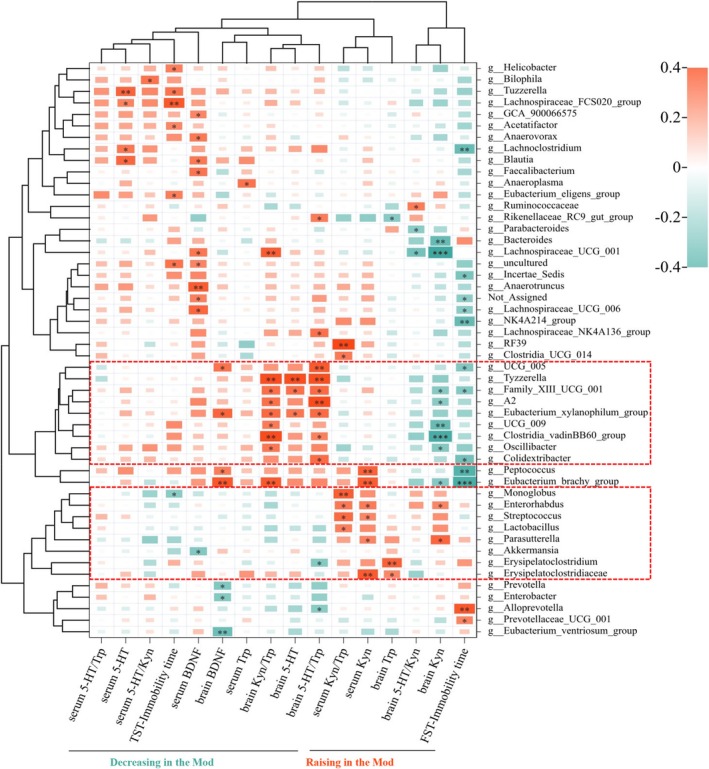
Correlation of gut microbiota and bio‐indexes of depressive mice. Gut microbiota at the genus level is clustered by the tested biochemistry indexes among all the sequenced samples, and Spearman's correlation heatmap is shown. Red/Green indicates that altered indexes are positively/negatively correlated with perturbed gut microbiota. *p < 0.05, **p < 0.01, ***p < 0.001.

In addition, we determined the genus level gut microbiota associated with depression by correlating the sequenced intestinal flora data with their bio‐indices. As shown in the correlation heatmap, the cluster analysis of depression‐related indicators reveals that the study's positively and negatively correlated indicators with depression can be aggregated on the same side (Figure 5 and Figure S7). Nine genera of bacteria were negatively associated with depression, as indicated by their correlation with depression‐related indicators. Conversely, eight genera exhibited a positive association with depression. Furthermore, three biomes, *g_Family_XIII_UCG_001, g_Clostridia_vadinBB60_group*, and *g_Lactobacillus*, exhibited significant intergroup variations in the aforementioned finding. The LPS induction resulted in a decrease in the levels of *g_Family_XIII_UCG_001* and *g_Clostridia_vadinBB60_group*. Conversely, it led to an increase in the level of *g__Lactobacillus*. The trend of these genera was effectively mitigated through the implementation of complexes (Figure 4J). Hence, we formulated the hypothesis that the nutrient‐synbiotic complex could enhance the composition of the gut microbial community by decreasing the abundance of *g__Lactobacillus* and increasing the levels of *g__Family_XIII_UCG_001* and *g__Clostridia_vadinBB60_group*. Such an effect would counteract the depressive‐like behavioral changes induced by LPS in mice.

## 4 Discussion

**FIGURE 6:**
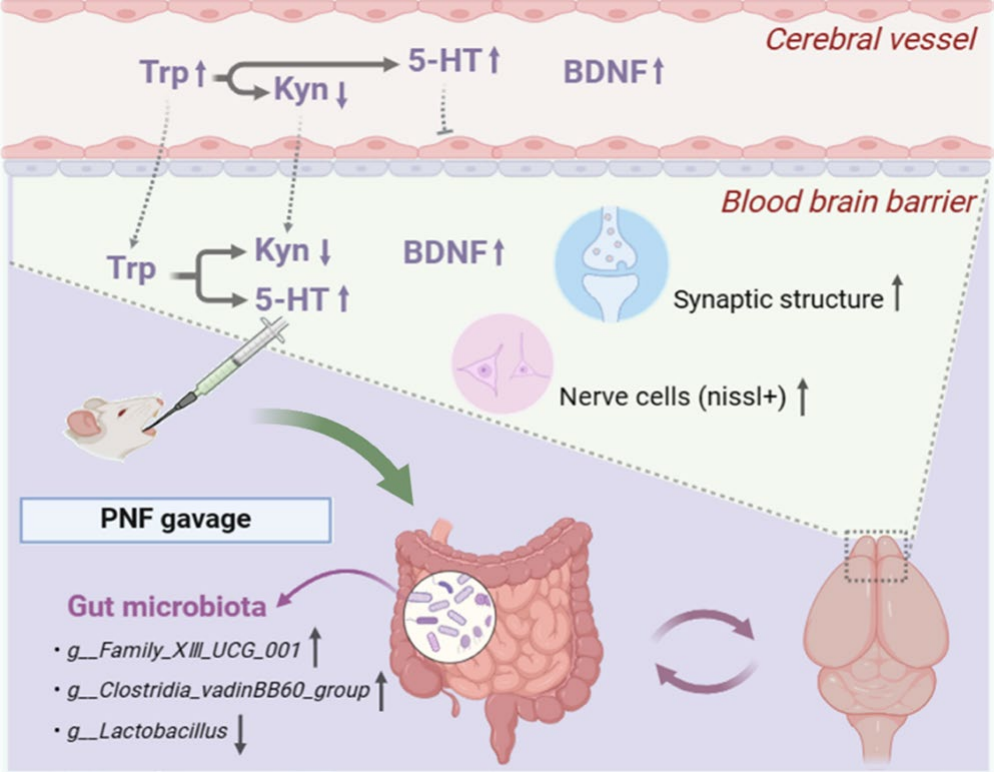
Schematic diagram of nutrient‐synbiotic complex to relieve depressive‐like behavior stimulated by LPS. 5‐HT, 5‐hydroxytryptamine; BDNF, brain‐derived neurotrophic factor; Kyn, kynurenic; LPS, lipopolysaccharide; Trp, tryptophan.

In the current study, the complex interventions, especially the nutrient‐synbiotic complex (Uni group), significantly reduced the depressive‐like behavioral alterations that were induced by LPS treatment. Additionally, the complex interventions reversed abnormal synaptic structure and neuronal damage induced by LPS in the PFC. The potential mechanisms involve the restoration of metabolic disorders in the tryptophan pathway and BDNF depletion. The development of depressive‐like symptoms could also be attributed to alterations in the structure of the intestinal flora in mice. However, the complex interventions successfully averted the detrimental effects of LPS exposure on the structure of the intestinal flora in mice. Through subsequent analysis, we postulated that the complexes could have ameliorated the depressive‐like behavioral changes induced by LPS by up‐regulating the levels of *g__Family_XIII_UCG_001* and *g__Clostridia_vadinBB60_group*, and down‐regulating the levels of *g__Lactobacillus*. The schematic diagram was shown in Figure 6.

Multiple intraperitoneal injections of LPS were employed in this study. The results demonstrated that LPS significantly induced avoidance behaviors in mice during OFT and EPM. Likewise, LPS‐induced despairing behavior in mice by prolonging their immobility time in the FST and TST. Notably, the complex interventions had the potential to rectify the depressive‐like behavioral changes induced by LPS in mice. Nevertheless, there was no significant difference in SPT, which was likely due to the non‐persistent depressive states or the interference of individual differences among the mice. SPT may require longer LPS exposure or the exclusion of mice that are not sensitive to sucrose preference. Comparable behavioral outcomes were observed in a recent study into the mechanisms through which BPA induces depressive‐like behavior (Wang et al. 2023).

It has long been believed that abnormal synaptic transmission and synaptic plasticity underlie cognitive dysfunction and emotional behavior (Cha et al. 2016; Stiles and Jernigan 2010). According to an early study, chronic unpredictable mild stimulation (CUMS) decreased synaptic strength and neurotransmission efficiency in neural circuits by altering synaptic ultrastructure in the rat hippocampus, increasing synaptic gap width, and decreasing PSD length and thickness (Li et al. 2015). The present study's findings revealed that the PFC of mice exposed to LPS exhibited ultrastructural damage, including synaptic loss, increased posterior membrane curvature, PSD thinning, and synaptic gap widening. Whereas all groups demonstrated a significant amelioration of the aforementioned synaptic alterations subsequent to the application of complexes. A number of recent studies related to depression have also yielded consistent electron microscopy findings (Wang et al. 2022; Yu et al. 2019). Furthermore, the alteration of synaptic plasticity in this study may be related to the change of BDNF levels. In the mammalian brain, BDNF can mediate various dependent processes, such as neuronal growth and differentiation, synapse formation and plasticity, and higher cognitive functions (Park and Poo 2013). Numerous animal studies and clinical evidence indicate that a deficiency in BDNF may induce depression by impairing the function of neurons in the brain, including those in the PFC and hippocampus (Gliwińska et al. 2023), which is consistent with our findings.

The symbiotic ecosystem, comprised of the gut microbial community, maintains homeostasis in the human body. The gut microbiota has emerged as a key modulator of brain physiology and pathology (Qiao et al. 2024). A growing number of studies indicated that the gut microbial composition of individuals with MDD was significantly altered compared to healthy individuals, particularly in terms of the diversity of gut microbes and the relative abundance of specific bacterial taxa. These results confirmed the association between dysbiosis of gut ecology and depression (Liu et al. 2022; Nikolova et al. 2021; Simpson et al. 2021). In this study, LPS significantly decreased the alpha‐diversity index (Chao1 and ACE) and altered the Beta‐diversity of mice, indicating the reduced abundance and diversity of gut microbes by LPS. However, the complexes could be beneficial in stabilizing this intestinal bacterial diversity and maintaining intestinal ecological balance.

To determine which specific flora level changes caused the microbial diversity changes, we used LEfSe analysis and multiple comparisons to examine the variations in the intestinal flora of each group at the phylum and genus levels. Eleven microbiomes with significant differences between the groups at the genus level were screened. The correlation analysis ultimately eliminated three bacterial groups (*g__Family_XIII_UCG_001, g__Clostridia_vadinBB60_group*, and *g__Lactobacillus*) that exhibited a positive or negative trend in relation to the primary depression outcome indicators. Among them, *g__Clostridia_vadinBB60_group* is a member of *f__Clostridiaceae* of *p__Thick‐walled Bacteria*. Recent studies have indicated that *g__Clostridia_vadinBB60_group* may serve as an indicator of gut‐brain axis health (Chin Fatt et al. 2023). The abundance of *f__Clostridia vadinBB60* was found to be lower in depressed patients compared to healthy controls (Liu et al. 2020). In addition, a mouse microbiome‐based study demonstrated that *Candida rugosa* lipase treatment increased the abundance of *g__Clostridia vadinBB60* in the intestine while reducing Alzheimer's disease‐like pathology (Menden et al. 2022). While the majority of *g__Clostridia_vadinBB60_groups* remain unclassified and play insignificant roles in metabolism and the microbiome, these taxa have been associated with an elevation in brainstem dopamine metabolites or 5‐HT precursors (5‐HIAA) (O'Connor et al. 2019; Ty et al. 2022). Consistent with the results of the aforementioned study concerning the *g__Clostridia_vadinBB60_group*, the current study investigated the effect of LPS treatment on the abundance levels of this genus, which was observed to be substantially decreased in the Mod group. Furthermore, a subsequent correlation analysis revealed a negative association between this genus and depression‐related metrics. Future studies should validate whether these genera directly modulate tryptophan metabolism via microbial‐derived metabolites (e.g., short‐chain fatty acids). The *g__Family_XIII_UCG_001*, which is also classified as *f__Clostridiaceae* within *p__Thick‐walled Bacteria*, exhibited a decrease in expression. In certain animal studies, the *g__Family_XIII_UCG‐001* was discovered to positively correlate with sugar‐water preference rates and negatively correlate with depressive‐like behaviors in the gut of mice exposed to chronic social defeat stress (CSDS) (Juckel et al. 2021). Similarly, the abundance of *g_*_*Family_XIII_UCG‐001* was notably reduced in mice that exhibited a chronic colitis model (Xu et al. 2021). This finding implies that the proliferation of this genus might be associated with inflammation onset. Given the paucity of research on this taxon, more studies are required to determine how it exerts its antidepressant effects. Moreover, among the three genera mentioned above, only *g__Lactobacillus*, which is classified as *f__Lactobacillaceae* within *p__Thick‐walled Bacteria*, was found to be upregulated in the Mod group. Interestingly, *f__Lactobacillaceae* and *Lactobacillus* spp. are commonly used as probiotic constituents that are ingested to regulate intestinal function; this association should be accompanied by beneficial effects. However, the current study revealed that the abundance of these microbes was significantly higher in the Mod compared to the Con, indicating that this genus was more actively expressed in response to LPS induction. According to a meta‐analysis, *g__Lactobacillus* tends to be enriched in MDD patients (Nikolova et al. 2021). This might be because certain strains may exert strain‐specific effects. The consumption of *Lactobacillus intestinalis* , *Lactobacillus reuteri* , and *Lactobacillus helveticus* has been linked in two studies to phenotypes of depression and pleasure deficiency, as well as a reduction in social behavior (Partrick et al. 2021; Wang et al. 2020). Consequently, these results demonstrate that LPS‐induced depressive‐like behavioral changes in mice are accompanied by a decrease in the abundance of two *Clostridiaceae* (*g__Family_XIII_UCG_001* and *g__Clostridia_vadinBB60_group*) that maintain stable intestinal function and an increase in the abundance of certain *Lactobacillus* spp. in the gut, which may have pathogenic effects.

Taken together, our formulation aligns with human‐safe doses of probiotics (CFU/day) and recommended dietary allowances of nutrients, suggesting feasibility for clinical trials. The antidepressant effects of complexes may be mediated through the regulation of gut homeostasis via the modulation of specific genera' abundance levels and the diversity of gut microbial communities. Nevertheless, there are some limitations in this study. First, LPS models acute inflammation, chronic models (e.g., CUMS) may better replicate depression. Second, while 16S rRNA sequencing identified significant alterations in gut microbiota composition at the genus level, the resolution of this method was insufficient to characterize species‐level dynamics. Finally, the nutrient‐synbiotic complex demonstrated significant antidepressant efficacy in murine models, though its translational potential for humans requires validation through randomized controlled trials (RCTs).

## 5 Conclusions

The nutrient‐synbiotic complex significantly reduces the depressive‐like behavioral changes in mice induced by LPS. It also reverses pathological alterations including synaptic loss and neuronal damage. Through modulating the proportions and levels of key active substances (Trp, Kyn, and 5‐HT) in the tryptophan metabolic pathway, the underlying mechanism may link to improving the metabolic homeostasis of depressed mice. In addition, the nutrient‐synbiotic complex ameliorates the reduction in gut microbial diversity induced by LPS‐induced depression in mice. Likewise, the nutrient‐synbiotic complex promotes the stabilization of gut microbial community structure by increasing the abundance levels of *g__Family_XIII_UCG_001* and *g__Clostridia_vadinBB60_group*, while decreasing the abundance level of *g__Lactobacillus*. Further research is warranted to develop a composite complex for clinical trials aimed at preventing and mitigating the onset and progression of depressive symptoms.

## Author Contributions

**Zhipeng Liu:** conceptualization (equal), writing – original draft (equal). **Shengchao Shi:** data curation (equal), validation (equal), visualization (equal). **Xiaoyu Zhang:** formal analysis (equal), methodology (equal). **Chao Wu:** data curation (equal), validation (equal). **Qin Yang:** data curation (equal). **Simeng Ren:** formal analysis (equal). **Yujuan Shan:** conceptualization (equal), supervision (equal). **Guanqiong Na:** conceptualization (equal), methodology (equal).

## Ethics Statement

Animal procedures in this work followed the Arrive guidelines. The animal experiments were approved by the Animal Ethics Committee of Wenzhou Medical University (Approved No. XMS9022‐0635).

## Conflicts of Interest

The authors declare no conflicts of interest.

## Supporting information


Appendix S1.

Data S1.

## Data Availability

All raw sequences were deposited in the NCBI Sequence Read Archive for microbiome data under Bioproject PRJNA1280655. The other datasets generated and analyzed during this study are included in Appendix S1 and Data S1.
