# Supplementary material for: Nutrient‐Synbiotic Complex Ameliorates LPS‐Induced Depressive‐Like Behavior via Modulation of Gut Microbiota and Tryptophan Metabolism in Mice
Source: Food Sci Nutr. 2025 Jul 15;13(7):e70628. doi: 10.1002/fsn3.70628 (PMC12263506; doi:10.1002/fsn3.70628)
Supplement: Supplementary file 1 — Appendix S1. [file FSN3-13-e70628-s002.docx]

**Nutrient-synbiotic complex** **ameliorates LPS-induced depressive-like behavior via** **modulation of gut microb****iota and tryptophan metabolism in mice**

Zhipeng Liu ^a, b^, Shengchao Shi ^a^, Xiaoyu Zhang ^a^, Chao Wu ^a^, Qin Yang ^a^, Simeng Ren ^c^，Yujuan Shan ^a, b^* and Guanqiong Na ^a, b^*

^a^ School of Public Health, Wenzhou Medical University, Wenzhou 325035, China.

^b^ Cixi Biomedical Research Institute, Wenzhou Medical University, Cixi 315300, China.

^c^ Department of Psychology, College of Liberal Arts, Wenzhou-Kean University, Wenzhou 325060, China.

* Corresponding authors: Guanqiong Na, E-mail: ngq@wmu.edu.cn,

Yujuan Shan, E-mail: [yujuanshan@wmu.edu.cn](mailto:yujuanshan@wmu.edu.cn).

**Supplementary Note 1**

Lipopolysaccharide (LPS), being a lipophilic molecule, is capable of penetrating the brain via a healthy or compromised blood–brain barrier (BBB). Depression may be exacerbated as a result of systemic LPS injection-induced depressive-like behavior characterized by behavioral alterations resembling those of acute systemic inflammation or infection (Maes et al., 2012). In fact, inflammation-related depressive symptoms in mice are frequently induced via recurrent injections of LPS (J. Liu et al., 2020). Similar to the symptoms observed in clinically depressed patients, LPS-induced pathological behaviors include reduced food consumption, sleep disruptions, heightened pain sensitivity, and diminished delight (Graeber & Streit, 2010). Therefore, current animal models induced with LPS are applicable to the screening of potential antidepressants and antianxiety drugs, in addition to their utility in research endeavors (Arioz et al., 2019; M. Li et al., 2017; W. Li et al., 2021).

**Supplementary Note 2**

Behavioral tests can be used to assess depressive-like behavior changes, such as Open Field Test (OFT) and Elevated Plus-maze Test (EPM) for evaluating spontaneous and exploratory behaviors in rodents, Tail Suspension Test (TST) and Forced Swimming Test (FST) for assessing behavioral despair, and Sucrose Preference Test (SPT) for quantifying indices of pleasure deficit (Hao et al., 2019; M.-Y. Liu et al., 2018).

**1. Open Field Test**

The OFT setup was consisted of a dark gray square box (50 × 50 × 40 cm^3^, length × width × height [lwh]). Light distribution within the box was ensured to be uniform, with no black corners present. Each mouse was initially confined to a corner of the open field device, with its back directed towards the central region. Subsequently, it was granted unrestricted mobility for 2 min in order to acclimate to the open field environment. The debugging software of SMART (v3.0.03), an animal behavioral analysis system, was then used to record the activity path of the mice. To assess the depressive-like behaviors exhibited by the mice, the analysis focused on the frequency of their entrances, stays, and travels within the central area.

**2. Elevated Plus-maze Test**

The EPM is comprised of two open arms (30 × 5 × 15 cm^3^, lwh) and two closed arms (30 × 5 × 15 cm^3^, lwh) that are crossed in a cross formation. The cross section of the central area measures 5 × 5 cm^2^. The vertical dimension of the labyrinth is 50 cm above the ground. Each mouse was cautiously positioned in the center of the labyrinth, facing the closed arms, at the onset of the experiment. A total of 6 min of experiments was documented with the SMART (v3.0.03). The anxiety behavior was determined by observing the duration and distance that the central portion of mouse’s body remained in the open and closed arms over the last 4 min.

**3. Tail Suspension Test**

TST employs a black tail suspension test box (25 × 25 × 40 cm^3^, lwh) in which mice are medical taped to a hook positioned 1.5 cm from the tip of their tails. This arrangement ensures that the mice remain approximately 15 cm above the ground and prevents their front feet from making contact with it. As measured by the duration of time the mice remained immobile with the exception of respiration, immobilization time was calculated. The mice became more despondent as the immobilization time increased. Using the SMART (v3.0.03), the activity of the rodents was monitored for 6 min, and the final 4 min of immobility were analyzed. The tail tapes were promptly removed and the mice were returned to their respective cages at the end of experiment.

**4. Forced Swimming Test**

FST is performed utilizing a transparent plastic cylinder with a 25 cm diameter and a 40 cm height. The mice were observed to struggle in the water after the cylinder was pre-filled with water (25 ± 1°C) at a height of approximately 25 cm to prevent them from reaching the bottom. Furthermore, it was noted that a black baffle should be erected between every two rodents to prevent mutual interference. Utilizing the SMART (v3.0.03), the activity of the mice for 6 min was documented, and the duration of immobility was analyzed in the final 4 min. The mice were promptly removed, dried, and returned to their respective cages at the conclusion of the experiment.

**5. Sucrose Preference Test**

Single-cage mice were acclimated for 24 h to a 1% sucrose solution (w/v) supplemented with potable water. The mice were then fasted for 12 h at night, and on the final day, they were duly instructed to perform the SPT tests for 24 hours. To prevent positional preference, the drinking bottles were repositioned for a 12-hour period during the day. To determine the sugar-water consumption over 24 h, the weight of drinking water bottles in each cage was weighed both prior to and subsequent to the experiment, and then Sucrose Preference Rate (SPR) was calculated. The SPR was calculated using the following formula:

SPR (%) = sucrose consumption/(sucrose consumption + water consumption) × 100%.

**Supplementary Note 3**

Tryptophan metabolism mainly includes two pathways, namely kynurenine (Kyn) pathway and 5-hydroxytryptophan (5-HT) pathway. The Kyn pathway comprises approximately 95% of the tryptophan metabolic pathway (M et al., 2019). Kyn is synthesized via the catalytic processes of tryptophan 2,3-dioxygenase (TDO) and indoleamine 2-3-dioxygenase (IDO1 or IDO2). Kyn is subsequently primarily converted to 3-hydroxykyn (3-HK) via kynurenine 3-monooxygenase (KMO). 3-HK is converted to xanthurenic acid (XA) and 3-hydroxyanthranilic acid (3-HAA) via the enzyme kynurenine aminotransferase (KAT) and kynureninase (Kynu), respectively. Next, 3-HAA is metabolized by aminocarboxymuconate-semialdehyde decarboxylase (ACMSD) to picolinic acid (PicA), which is then non-enzymatically converted to quinolinic acid (QUIN). The enzyme quinolinic acid phosphoribosyl transferase (QPRT) subsequently converts QUIN to NAD+. Kyn is additionally metabolized to a lesser degree by the enzymatic activity of KAT into kynurenic acid (KA) (Savitz, 2020). Regarding the 5-HT pathway, tryptophan hydroxylase catalyzes the conversion of tryptophan into 5-hydroxytryptophan (5-HTP). An aromatic amino acid decarboxylase (AADC) subsequently catalyzes the conversion of 5-HTP to 5-HT. Following that, 5-HT can be metabolized by N-acetylserotonin O-methyltransferase (ASMT) to melatonin (a neurohormone) or by the enzyme monoamine oxidase (MAO) to produce 5-hydroxyindoleacetic acid (5-HIAA), the principal metabolite of 5-HT (Höglund et al., 2019).

The pathogenesis of depression is significantly influenced by dysregulated Trp metabolism, which involves the major Trp metabolic pathways including Kyn and 5-HT (Colle et al., 2020; Marx et al., 2021; Wang et al., 2022). 5-HT is one of the most essential neurotransmitters in the human body and is implicated in behavioral and emotional response regulation. The study revealed that MDD patients exhibited considerably diminished plasma levels of Trp and 5-HT in comparison to the healthy individuals (Colle et al., 2020; Marx et al., 2021) . Conversely, Kyn has the potential to induce additional neuronal injury via the synthesis of toxic metabolites, including quinolinic acid and 3-hydroxykynurenine, which are involved in the pathogenesis of psychiatric conditions like depression (Brown et al., 2021; Deng et al., 2021). Previous studies have reported depressed mice has been associated with decreased 5-HT levels and increased Kyn levels (Deng et al., 2021).

**Reference:**

Arioz, B. I., Tastan, B., Tarakcioglu, E., Tufekci, K. U., Olcum, M., Ersoy, N., Bagriyanik, A., Genc, K., & Genc, S. (2019). Melatonin Attenuates LPS-Induced Acute Depressive-Like Behaviors and Microglial NLRP3 Inflammasome Activation Through the SIRT1/Nrf2 Pathway. *Frontiers in Immunology*, *10*, 1511. https://doi.org/10.3389/fimmu.2019.01511

Brown, S. J., Huang, X.-F., & Newell, K. A. (2021). The kynurenine pathway in major depression: What we know and where to next. *Neuroscience and Biobehavioral Reviews*, *127*, 917–927. https://doi.org/10.1016/j.neubiorev.2021.05.018

Colle, R., Masson, P., Verstuyft, C., Fève, B., Werner, E., Boursier-Neyret, C., Walther, B., David, D. J., Boniface, B., Falissard, B., Chanson, P., Corruble, E., & Becquemont, L. (2020). Peripheral tryptophan, serotonin, kynurenine, and their metabolites in major depression: A case-control study. *Psychiatry and Clinical Neurosciences*, *74*(2), 112–117. https://doi.org/10.1111/pcn.12944

Deng, Y., Zhou, M., Wang, J., Yao, J., Yu, J., Liu, W., Wu, L., Wang, J., & Gao, R. (2021). Involvement of the microbiota-gut-brain axis in chronic restraint stress: Disturbances of the kynurenine metabolic pathway in both the gut and brain. *Gut Microbes*, *13*(1), 1–16. https://doi.org/10.1080/19490976.2020.1869501

Graeber, M. B., & Streit, W. J. (2010). Microglia: Biology and pathology. *Acta Neuropathologica*, *119*(1), 89–105. https://doi.org/10.1007/s00401-009-0622-0

Hao, Y., Ge, H., Sun, M., & Gao, Y. (2019). Selecting an Appropriate Animal Model of Depression. *International Journal of Molecular Sciences*, *20*(19), 4827. https://doi.org/10.3390/ijms20194827

Höglund, E., Øverli, Ø., & Winberg, S. (2019). Tryptophan Metabolic Pathways and Brain Serotonergic Activity: A Comparative Review. *Frontiers in Endocrinology*, *10*, 158. https://doi.org/10.3389/fendo.2019.00158

Li, M., Li, C., Yu, H., Cai, X., Shen, X., Sun, X., Wang, J., Zhang, Y., & Wang, C. (2017). Lentivirus-mediated interleukin-1β (IL-1β) knock-down in the hippocampus alleviates lipopolysaccharide (LPS)-induced memory deficits and anxiety- and depression-like behaviors in mice. *Journal of Neuroinflammation*, *14*(1), 190. https://doi.org/10.1186/s12974-017-0964-9

Li, W., Ali, T., He, K., Liu, Z., Shah, F. A., Ren, Q., Liu, Y., Jiang, A., & Li, S. (2021). Ibrutinib alleviates LPS-induced neuroinflammation and synaptic defects in a mouse model of depression. *Brain, Behavior, and Immunity*, *92*, 10–24. https://doi.org/10.1016/j.bbi.2020.11.008

Liu, J., He, Y., Cheng, K., & Xie, P. (2020). Changed PGA and POSTN levels in choroid plexus are associated with depressive-like behaviors in mice. *Biochemical and Biophysical Research Communications*, *524*(1), 231–235. https://doi.org/10.1016/j.bbrc.2020.01.076

Liu, M.-Y., Yin, C.-Y., Zhu, L.-J., Zhu, X.-H., Xu, C., Luo, C.-X., Chen, H., Zhu, D.-Y., & Zhou, Q.-G. (2018). Sucrose preference test for measurement of stress-induced anhedonia in mice. *Nature Protocols*, *13*(7), 1686–1698. https://doi.org/10.1038/s41596-018-0011-z

M, P., Eaa, N., Uf, R., F, F., & Ca, O. (2019). Tryptophan metabolism as a common therapeutic target in cancer, neurodegeneration and beyond. *Nature Reviews. Drug Discovery*, *18*(5). https://doi.org/10.1038/s41573-019-0016-5

Maes, M., Berk, M., Goehler, L., Song, C., Anderson, G., Gałecki, P., & Leonard, B. (2012). Depression and sickness behavior are Janus-faced responses to shared inflammatory pathways. *BMC Medicine*, *10*, 66. https://doi.org/10.1186/1741-7015-10-66

Marx, W., McGuinness, A. J., Rocks, T., Ruusunen, A., Cleminson, J., Walker, A. J., Gomes-da-Costa, S., Lane, M., Sanches, M., Diaz, A. P., Tseng, P.-T., Lin, P.-Y., Berk, M., Clarke, G., O’Neil, A., Jacka, F., Stubbs, B., Carvalho, A. F., Quevedo, J., … Fernandes, B. S. (2021). The kynurenine pathway in major depressive disorder, bipolar disorder, and schizophrenia: A meta-analysis of 101 studies. *Molecular Psychiatry*, *26*(8), 4158–4178. https://doi.org/10.1038/s41380-020-00951-9

Savitz, J. (2020). The kynurenine pathway: A finger in every pie. *Molecular Psychiatry*, *25*(1), 131–147. https://doi.org/10.1038/s41380-019-0414-4

Wang, D., Wu, J., Zhu, P., Xie, H., Lu, L., Bai, W., Pan, W., Shi, R., Ye, J., Xia, B., Zhao, Z., Wang, Y., Liu, X., & Zhao, B. (2022). Tryptophan-rich diet ameliorates chronic unpredictable mild stress induced depression- and anxiety-like behavior in mice: The potential involvement of gut-brain axis. *Food Research International (Ottawa, Ont.)*, *157*, 111289. https://doi.org/10.1016/j.foodres.2022.111289

**Supplementary Table**

**Table S1 Formula component.**

| **Group Name** | **Component** |
| --- | --- |
| Control group (Con) | Maltodextrin |
| Model group (Mod) | Maltodextrin |
| Nutrients group (Nut) | Nutrient complex (vitamin B_12_, folic acid, selenium, iron and zinc) and maltodextrin |
| Prebiotics group (Pre) | Prebiotics (oligo-galactose and oligo-fructose) and maltodextrin |
| Probiotics group (Pro) | Probiotic strains (Bifidobacterium longum, Lactobacillus casei, Lactobacillus suis, Lactobacillus rhamnosus, Lactobacillus fermentum and Lactococcus lactis subspecies lactis) and maltodextrin |
| United group (Uni) | All the above |

Note. maltodextrin dose (4.6 mg/kg·bw) as a control.

**Table S2 LDA score and *P*-values of LEfSe analysis.**

| **Biomarker** | **Log10** | **Group** | **LDA score** | ***P*-value** |
| --- | --- | --- | --- | --- |
| *g__Family_XIII_UCG_001* | 1.8635 | NUT | 2.2963 | 0.0318 |
| *g__Peptococcus* | 2.4003 | NUT | 2.1390 | 0.0219 |
| *c__Bacilli* | 4.0875 | MOD | 3.7791 | 0.0132 |
| *o__Lactobacillales* | 3.8261 | MOD | 3.5932 | 0.0179 |
| *g__Lactobacillus* | 3.8187 | MOD | 3.5867 | 0.0184 |
| *f__Lactobacillaceae* | 3.8187 | MOD | 3.5867 | 0.0184 |
| *c__Gammaproteobacteria* | 3.7046 | MOD | 3.3268 | 0.0384 |
| *f__Veillonellaceae* | 2.3443 | MOD | 2.9083 | 0.0269 |
| *o__Staphylococcales* | 2.9607 | MOD | 2.8543 | 0.0030 |
| *g__Staphylococcus* | 2.9607 | MOD | 2.7524 | 0.0030 |
| *g__Psychrobacter* | 1.2363 | MOD | 2.7464 | 0.0244 |
| *f__Staphylococcaceae* | 2.9607 | MOD | 2.7418 | 0.0030 |
| *g__Dialister* | 2.2862 | MOD | 2.6820 | 0.0238 |
| *f__Psychromonadaceae* | 1.2660 | MOD | 2.5802 | 0.0352 |
| *g__Psychromonas* | 1.2463 | MOD | 2.5198 | 0.0352 |
| *f__Pseudoalteromonadaceae* | 2.2351 | MOD | 2.4948 | 0.0295 |
| *g__Pseudoalteromonas* | 2.2351 | MOD | 2.4625 | 0.0295 |
| *g__Ruminococcaceae* | 2.4601 | MOD | 2.3805 | 0.0126 |
| *o__Alteromonadales* | 2.2977 | MOD | 2.3271 | 0.0295 |
| *c__Campylobacteria* | 4.0076 | CON | 3.6331 | 0.0172 |
| *p__Campilobacterota* | 4.0076 | CON | 3.6308 | 0.0172 |
| *f__Helicobacteraceae* | 4.0076 | CON | 3.6298 | 0.0194 |
| *o__Campylobacterales* | 4.0076 | CON | 3.6296 | 0.0172 |
| *g__Helicobacter* | 4.0076 | CON | 3.6281 | 0.0194 |

**Table S3 Comparison of different species at the genus level.**

| **Genus** | **Phylum** | ***P*-value** | ***P*.signif** | **MOD vs. CON** |
| --- | --- | --- | --- | --- |
| *g_Staphylococcus* |  | 0.000741 | *** | up |
| *Prevotellaceae_NA* |  | 0.00119 | ** | down |
| *g_Helicobacter* |  | 0.003783 | ** | down |
| *g_Agathobacter* |  | 0.004326 | ** | up |
| *g_Family_XIII_UCG-001* |  | 0.009231 | ** | down |
| *g_Dialister* |  | 0.009717 | ** | up |
| *g_Ruminococcaceae* |  | 0.019052 | * | ns |
| *g_Clostridia_vadinBB60_group* |  | 0.019211 | * | down |
| *g_Lactobacillus* |  | 0.03072 | * | up |
| *Lachnospiraceae_uncultured* |  | 0.031075 | * | down |
| *g_uncultured* |  | 0.03364 | * | down |
| *g_UCG-002* |  | 0.043451 | * | ns |
| *g_Muribaculum* |  | 0.047081 | * | down |

Note. ◼: Firmicutes; ◼: Bacteroidota; ◼: Campilobacterota; ◼: Proteobacteria. **^*^***P* < 0.05, **^**^***P* < 0.01 and **^***^***P* < 0.001.

**Supplementary Figure**

**
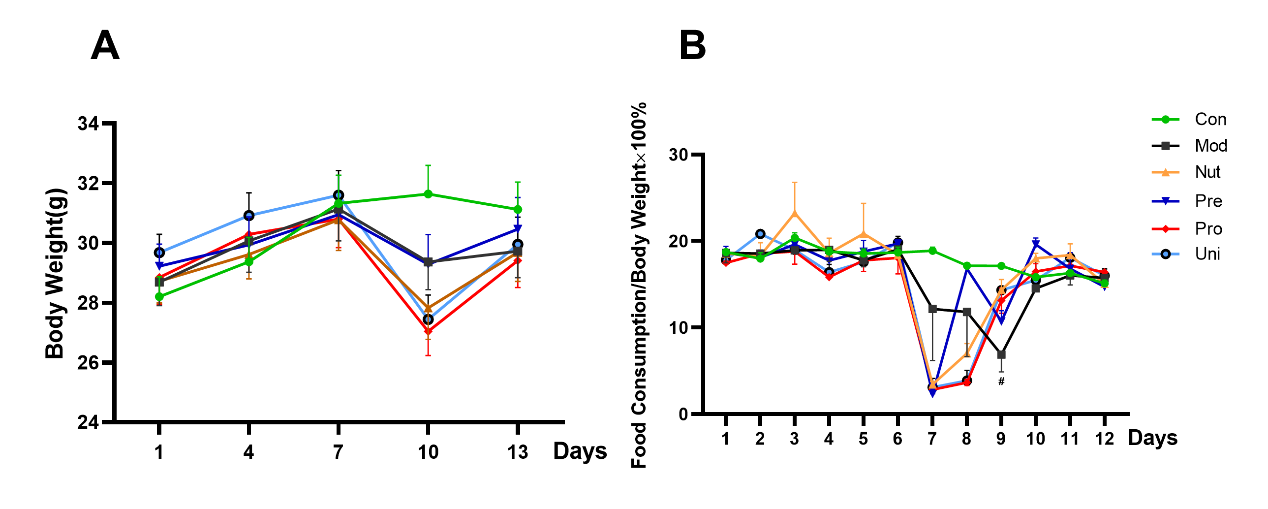
**

**Figure S1 Nutrient-synbiotic complex has no effect on their body weight and food consumption in LPS-induced models of depression**

*Note:* Body weight (A) and Food consumption (B) changes of each group mice at different time points. Data are presented as mean ± SEM, n = 8/group. ^#^*P* < 0.05 versus the Con. Significance was evaluated by Two‐way Repeated Measures ANOVA followed by LSD's multiple comparisons test. Con, control group; LPS, lipopolysaccharide; LSD, least significance difference; Mod, model group; Nut, nutrients group; Pre, prebiotics group; Pro, probiotics group; Uni, united group.

**
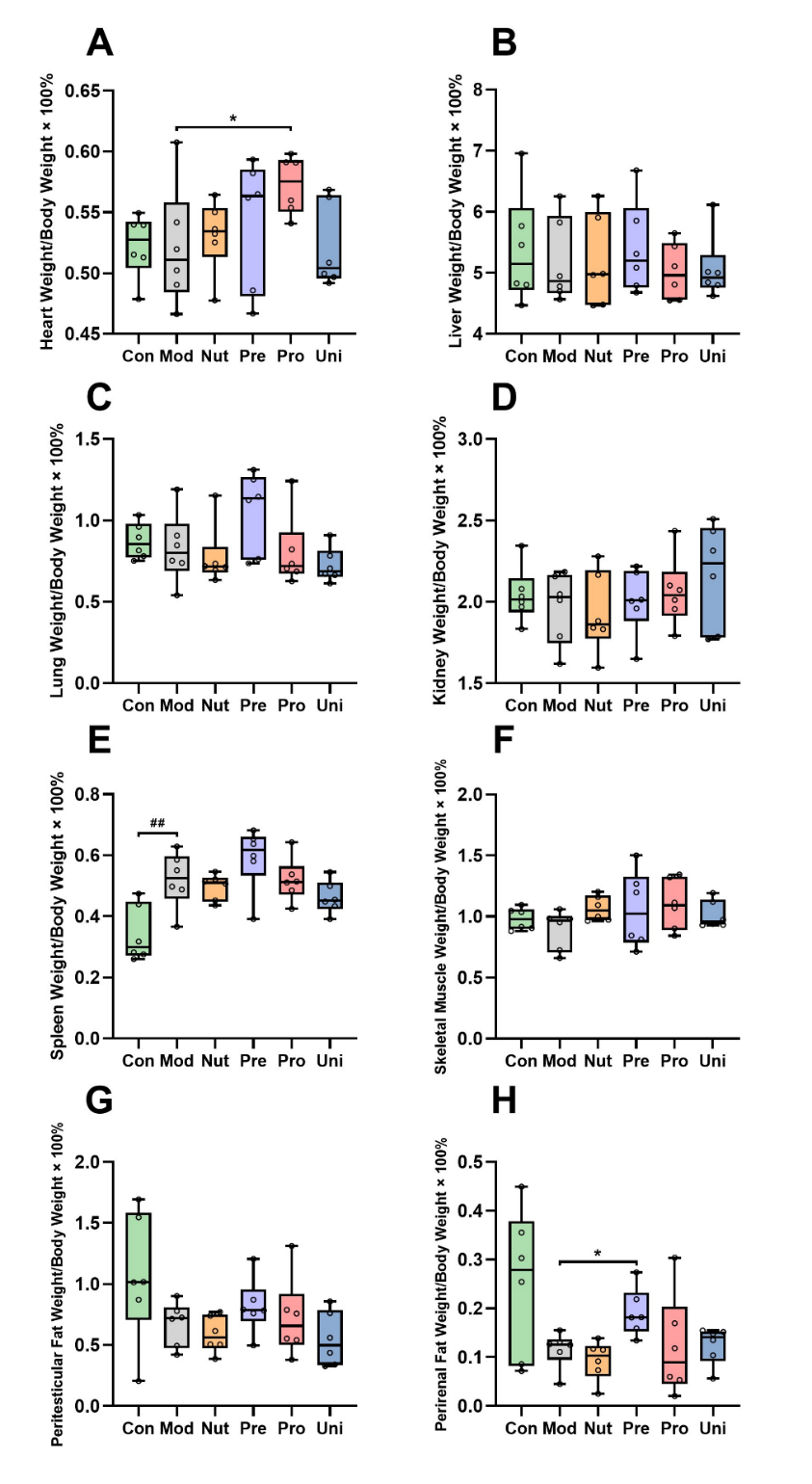
**

**Figure S2** **Nutrient-synbiotic complex has no effect on the majority of organ coefficients in LPS-induced models of depression**

*Note:* Heart (A), Liver (B), Lung (C), Kidney (D), Spleen (E), Skeletal muscle (F), Peritesticular fat (G) and Perirenal fat (H) coefficients’ changes of each group mice at different time points. The organ coefficient is equal to organ weight/body weight ×100%. Data are presented as mean ± SEM, n = 8/group. ^#^*P* < 0.05 versus the Con. Significance was evaluated by one‐way Repeated Measures ANOVA followed by LSD's multiple comparisons test. Con, control group; LPS, lipopolysaccharide; LSD, least significance difference; Mod, model group; Nut, nutrients group; Pre, prebiotics group; Pro, probiotics group; Uni, united group.


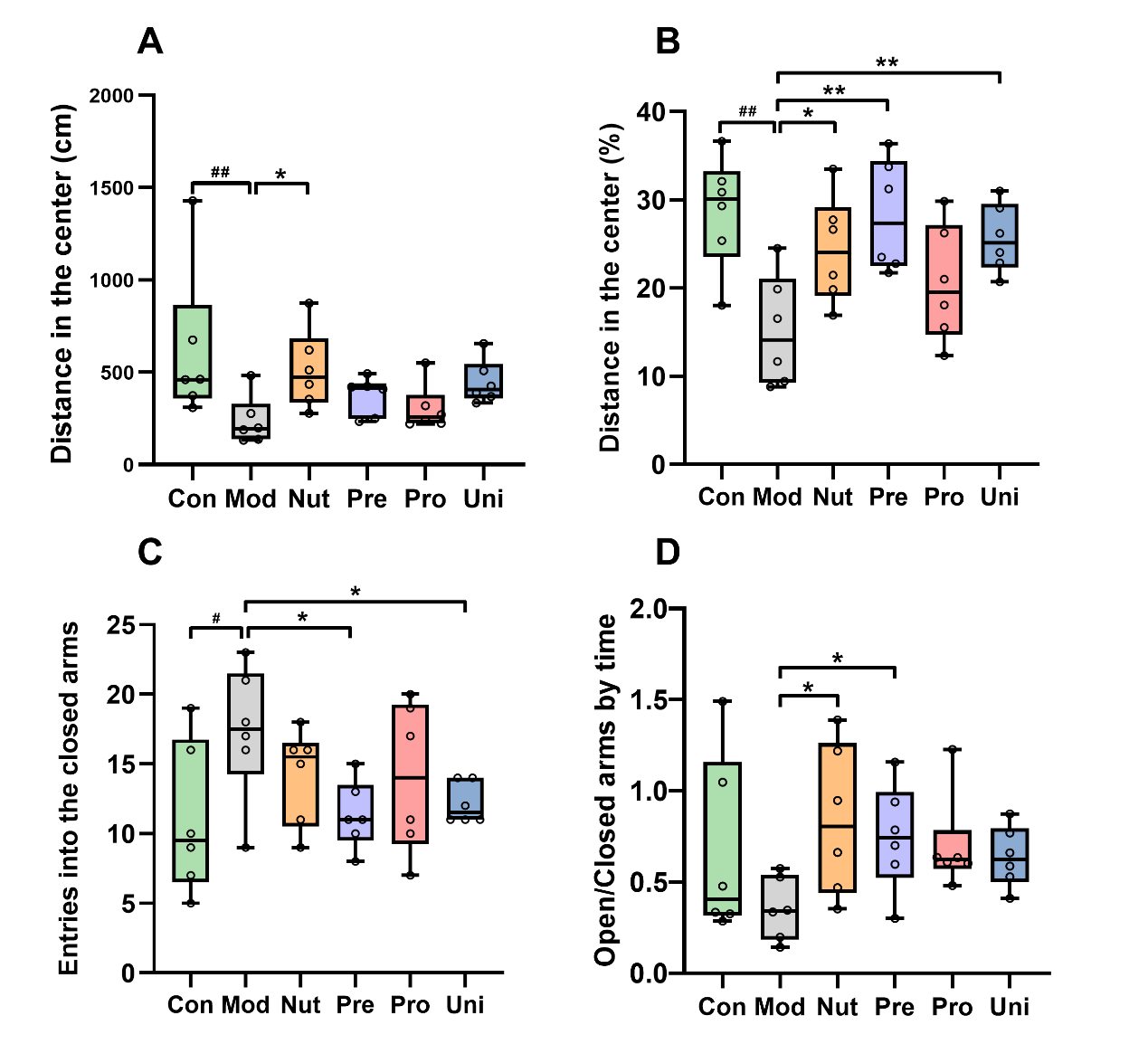


**Figure S3 Nutrient-synbiotic complex alleviates LPS-induced depressive-like behaviors**

*Note:* Quantification on absolute traveling distance in the center (**A**) and percentage of traveling distance in the center (**B**) of OFT. Quantification on entries into the closed arms (**E**) and ratio of time in open/closed arms (**F**) in the EPM, Data are presented as mean ± SEM, n = 6/group. ^#^*P* < 0.05 and ^##^*P* < 0.01 versus the Con; ^*^*P* < 0.05 and ^**^*P* < 0.01 versus the Mod. Significance was evaluated by one-ANOVA followed by LSD's multiple comparisons test. Con, control group; LPS, lipopolysaccharide; LSD, least significance difference; Mod, model group; Nut, nutrients group; Pre, prebiotics group; Pro, probiotics group; Uni, united group.

**
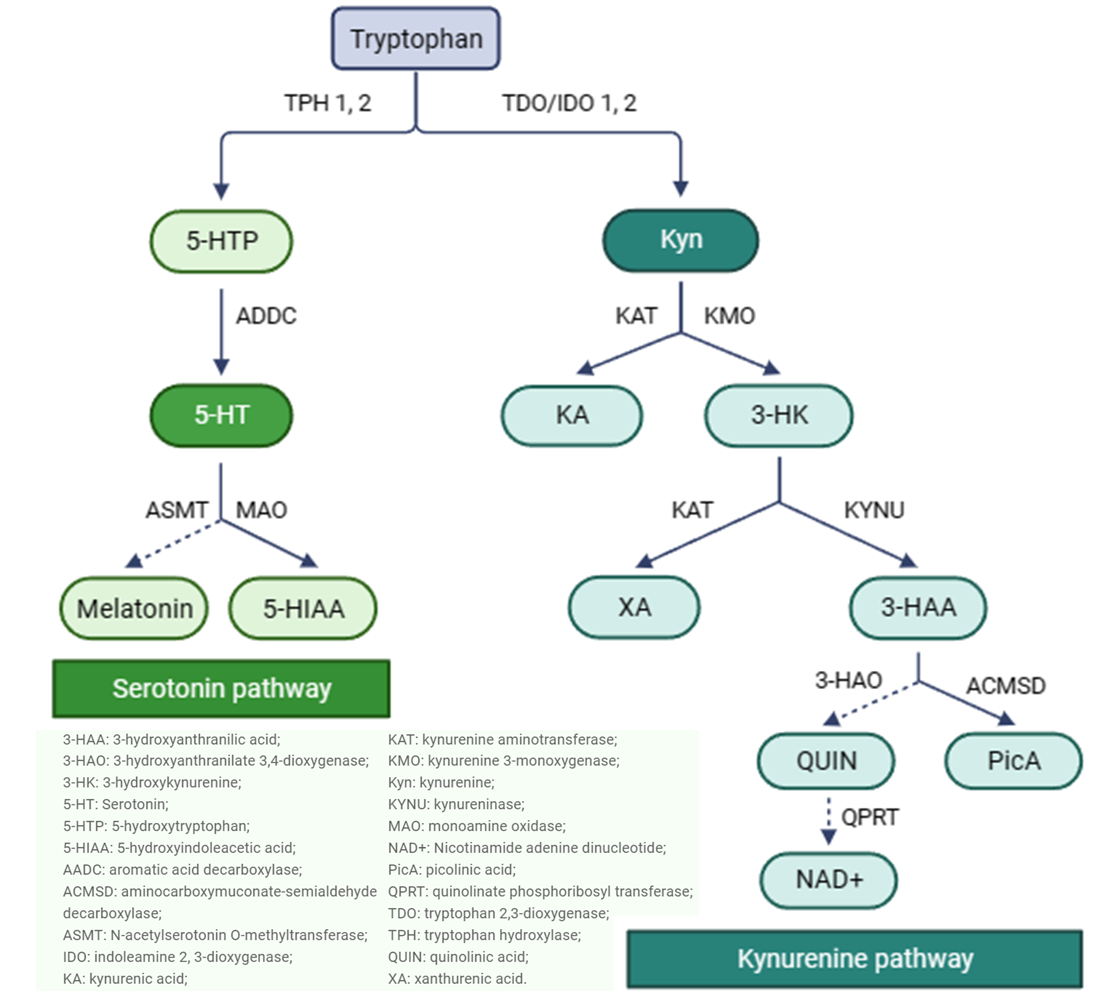
**

**Figure S4 Emotion regulation of tryptophan metabolites**

*Note:* Illustration created with BioRender (<https://biorender.com/>).


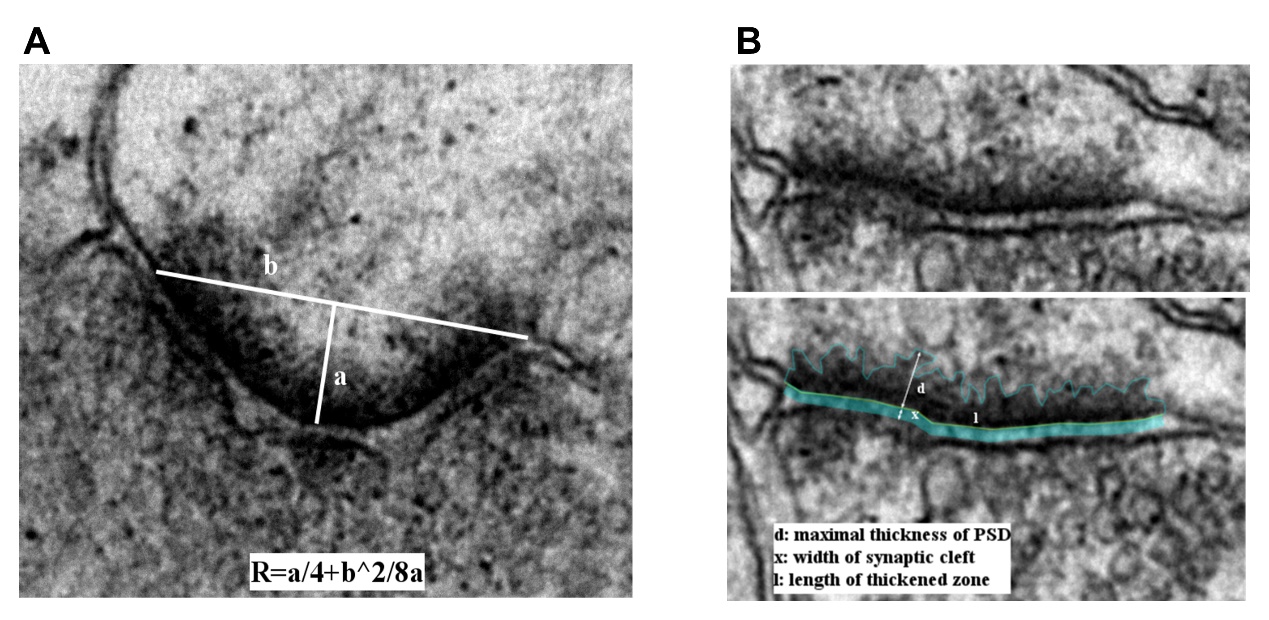


**Figure S5 Ultrastructure of synapses observed under TEM**

*Note:* (A) Radius of curvature of the postsynaptic thickening; (B) PSD thickness, width of synaptic cleft and length of thickened zone. In order to determine the mean and maximum values of their respective parameters, five equal parts were selected. PSD, postsynaptic density; TEM, transmission electron microscope.


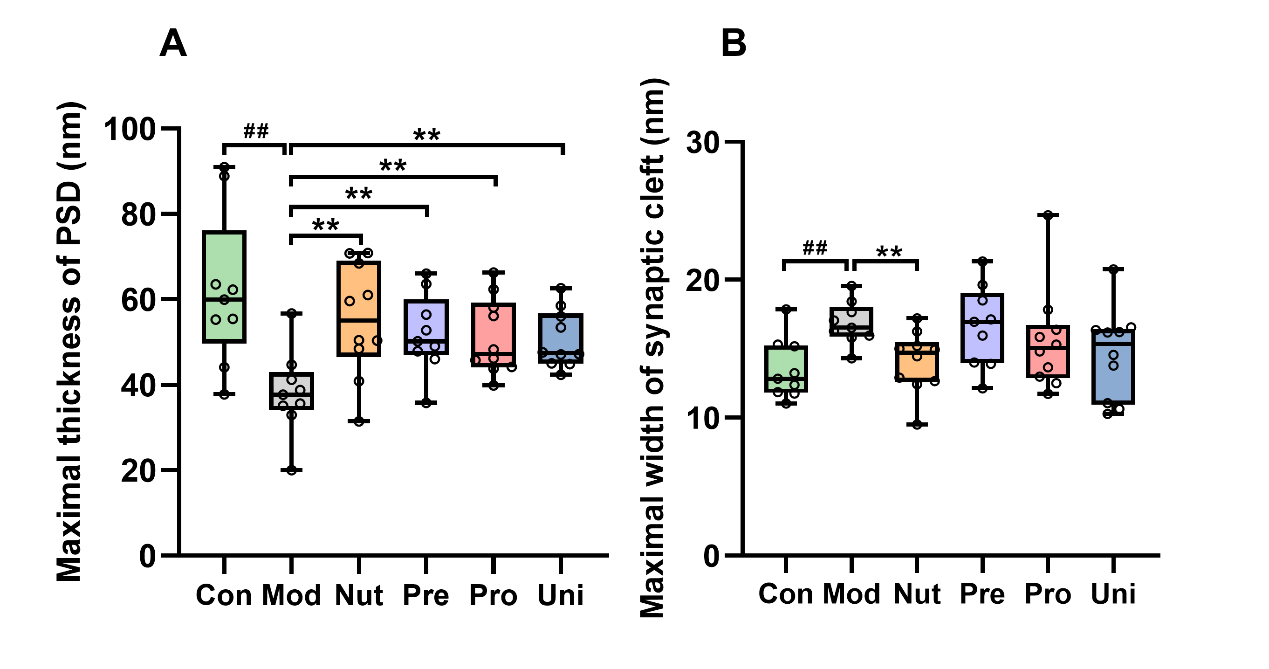


**Figure S6 Nutrient-synbiotic complex ameliorates synaptic plasticity alterations in the PFC induced by LPS**

*Note:* Quantitative analysis of maximal thickness of PSD (A) and maximal width of synaptic cleft (B) in PFC neurons. Data are presented as mean ± SEM, n = 6/group. ^##^*P* < 0.01 versus the Con; ^**^*P* < 0.01 versus the Mod. Significance was evaluated by one-ANOVA followed by LSD's multiple comparisons test. Con, control group; LPS, lipopolysaccharide; LSD, least significance difference; Mod, model group; Nut, nutrients group; Pre, prebiotics group; Pro, probiotics group; Uni, united group.


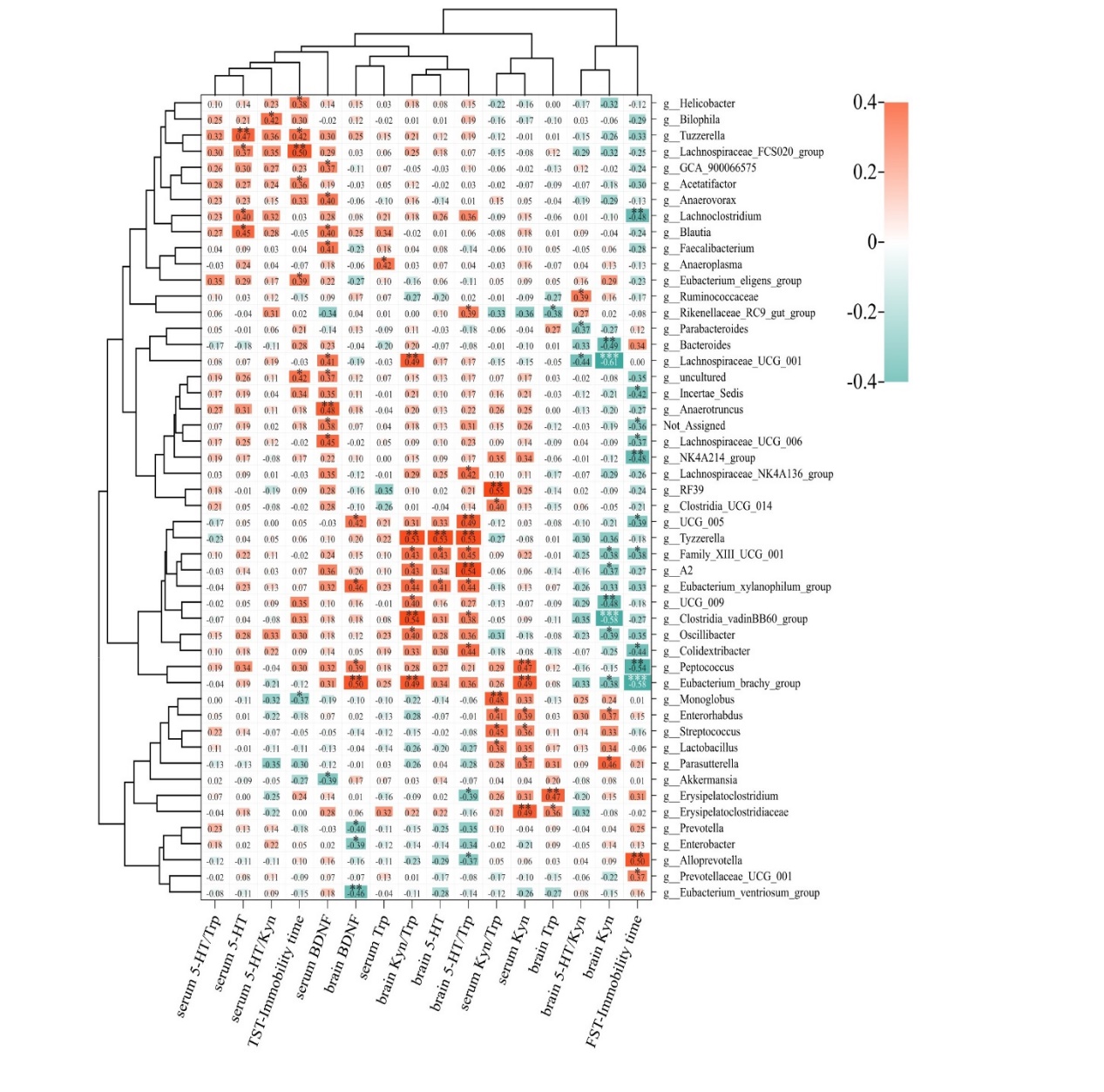

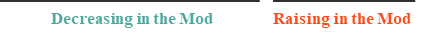


**Figure S7 Correlation of gut microbiota and bio-indexes of depressive mice,** **with numerical values**

*Note:* Gut microbiota at the genus level is clustered by the tested biochemistry indexes among all the sequenced samples. Spearman’s correlation heatmap with R-values is shown. Red/Green indicates that altered indexes are positively/negatively correlated with perturbed gut microbiota. ^*^*P* < 0.05, ^**^*P* < 0.01, ^***^*P* < 0.001.
